# Optimising Haematopoietic Stem Cell Transplantation: Enhancing Myeloablation Sensitivity and Alleviating Anaemia Using Roxadustat (FG‐4592)

**DOI:** 10.1111/cpr.70230

**Published:** 2026-07-10

**Authors:** Guanheng Yang, Jiahui Wu, Xinbing Guo, Wenchen Shen, Xiao Sang, Xiuli Gong, Shu‐Zhen Huang, Jingzhi Zhang, Fanyi Zeng

**Affiliations:** ^1^ Shanghai Institute of Medical Genetics, Shanghai Children's Hospital, Shanghai Jiao Tong University School of Medicine Shanghai China; ^2^ Department of Histology, Embryology, Genetics and Developmental Biology Shanghai Jiao Tong University School of Medicine Shanghai China; ^3^ NHC Key Laboratory of Medical Embryogenesis and Developmental Molecular Biology, Shanghai Key Laboratory of Embryo and Reproduction Engineering Shanghai China; ^4^ School of Pharmacy, Macau University of Science and Technology Macau China

**Keywords:** bone marrow microenvironment, haematopoietic stem cell, myeloablation, Roxadustat (FG‐4592), thalassaemia, transplantation

## Abstract

Haematopoietic stem cell transplantation (HSCT) is an effective therapy for a broad range of hematologic, autoimmune and genetic diseases. Successful HSCT requires adequate myeloablation to create space in the bone marrow microenvironment for donor haematopoietic stem and progenitor cells. Standard conditioning with busulfan and cyclophosphamide (BuCy), although widely used, may not completely clear recipient cells and can cause significant toxicity. Roxadustat (FG‐4592), a hypoxia‐inducible factor prolyl hydroxylase inhibitor used to treat renal anaemia, modulates HIF‐1α stability and influences erythropoiesis, iron metabolism and cellular stress responses. Here, we investigated whether co‐administration of FG‐4592 during BuCy conditioning improves myeloablation efficiency and enhances donor‐cell engraftment. Our results show that FG‐4592 increases the sensitivity of bone marrow mononuclear cell (BM‐MNC) populations to BuCy conditioning, promoting deeper depletion of both haematopoietic and non‐haematopoietic recipient cells. This improved clearance supports enhanced donor‐cell residency following transplantation. Furthermore, in *β* ^*654*^ thalassaemia mice, FG‐4592 alleviated anaemia and reduced splenic iron deposition after HSCT. These findings suggest that FG‐4592 can serve as an effective adjuvant to standard myeloablation regimens, supporting improved engraftment and hematologic recovery. To our knowledge, this is the first study demonstrating that a HIF‐PH inhibitor can enhance conditioning efficiency during HSCT.

## Graphical abstract

The use of FG‐4592 in the process of myeloablation can promote haematopoietic cell apoptosis for more effective myeloablation, maintain the number of RBC and may create a pluripotent microenvironment suitable for donor cells, so as to enable more effective haematopoietic reconstruction of donor cells in vivo.

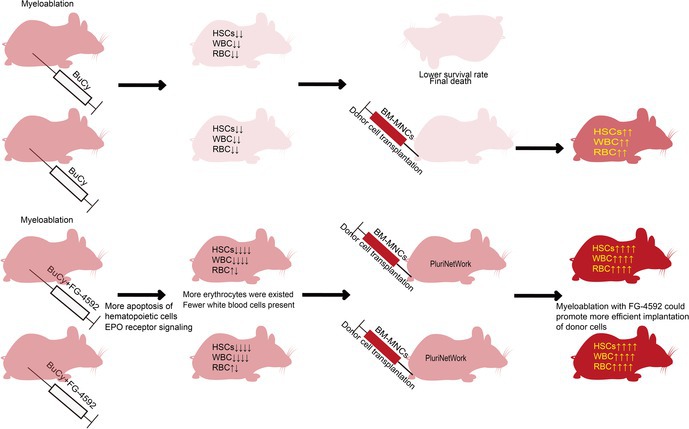

**Abbreviations**
BM‐MNCs: bone marrowmononuclear cells
IBMI: intra‐bone marrow injection
WT mice: wild‐type mice

## 1 Introduction

Haematopoietic stem cell (HSC) transplantation is an effective therapeutic strategy for numerous malignant hematologic disorders [1, 2, 3, 4]. A central determinant of successful transplantation is achieving adequate myeloablation, which eliminates recipient haematopoietic and immune cells and creates space within the marrow niche for donor HSC engraftment. Chemotherapy‐based regimens, particularly busulfan followed by cyclophosphamide (BuCy), remain the standard approach. The BuCy protocol was established in the 1980s at Johns Hopkins University for conditioning patients with acute lymphoblastic leukaemia [5, 6]. Despite its clinical utility, BuCy conditioning carries substantial toxicity. Busulfan is associated with veno‐occlusive disease (VOD) [7], whereas high‐dose cyclophosphamide can cause acute cardiotoxicity, endothelial injury, arrhythmias and fatal myopericarditis [8]. Combined BuCy treatment may also induce haemorrhagic cystitis and subclinical seizures [9], potentially compromising immune stability and increasing risks of infection or graft‐versus‐host disease (GVHD) [10, 11], while relapse after transplant remains life‐threatening [12]. These limitations underscore the need to enhance conditioning efficacy while reducing collateral tissue damage. Roxadustat is an orally active hypoxia‐inducible factor prolyl hydroxylase inhibitor (HIF‐PHI) that stabilises HIF‐1α and prevents its degradation [13]. It has demonstrated radioprotective activity in haematopoietic cells and protective effects in diverse tissue injury contexts, including glomerular endothelial damage in diabetic nephropathy [14, 15]. As the first approved drug in the HIF‐PHI class, roxadustat represents a major advance in anaemia therapy. Its hematologic benefits derive from HIF‐1α stabilisation, which stimulates erythropoietin (EPO) production and enhances iron metabolism [16, 17]. By increasing endogenous EPO levels and elevating haemoglobin [18], roxadustat effectively treats anaemia [19]. Clinical studies—including phase II trials [20], multicentre studies in China [21, 22] and phase III trials in Japan [23] have confirmed its efficacy in patients with chronic kidney disease, both dialysis‐dependent and non–dialysis–dependent.

Beyond anaemia management, roxadustat exerts cytoprotective and immunomodulatory effects relevant to transplantation. It protects against ischemia‐induced acute kidney injury by inducing CD73 and suppressing AIM2 inflammasome activation [24]. It promotes angiogenesis via HIF‐1α/VEGF/VEGFR2 signalling and accelerates diabetic wound healing [25]. Roxadustat may mitigate doxorubicin‐induced cardiotoxicity by upregulating HIF‐1α–responsive genes that counteract apoptosis and oxidative stress [26]. Additional studies have suggested that roxadustat enhances compensatory lung growth [27], promotes repair in DSS‐induced colitis [28], and modulates macrophage metabolism through HIF‐2α, thereby attenuating NLRP3‐mediated inflammation and insulin resistance [29]. Importantly, Dalby et al. demonstrated that perivascular stromal cells stably expressing HIF‐1α could effectively support haematopoietic reconstitution of long‐term repopulating HSCs without exogenous supplementation [30], highlighting a potential role for HIF stabilisation in improving marrow niche function.

Given the broad protective and haematopoietic effects of roxadustat, we hypothesised that administering roxadustat during myeloablation would mitigate conditioning‐associated injury, enhance recipient marrow microenvironment quality and improve donor‐cell engraftment. To test this, we co‐administered roxadustat (FG‐4592) with BuCy conditioning in wild‐type (WT) mice and evaluated haematopoietic recovery following donor BM‐MNC transplantation. We further assessed its therapeutic potential in *β* ^*654*^ thalassaemia mice, a model characterised by ineffective erythropoiesis and chronic anaemia.

This study demonstrates that FG‐4592 enhances the depth of myeloablation, improves recipient survival, promotes donor‐cell implantation, accelerates erythroid recovery and ameliorates anaemia and pathological haematopoiesis in the *β* ^*654*^ model. These findings support FG‐4592 as a promising adjunct to conventional conditioning regimens to optimise haematopoietic stem cell transplantation (HSCT) outcomes.

## 2 Materials and Methods

### 2.1 Recipient Mouse Preparation

C57BL/6J (CD45.2^+^) WT mice were obtained from the Shanghai Nanfang Research Center for Biomodel Organisms. The *β* ^*654*^ thalassaemia mice (CD45.2^+^), purchased from The Jackson Laboratory, carry one WT murine β‐major globin allele and one mutant human β^IVS‐2‐654^ allele caused by a C→T substitution at nucleotide 654 of intron 2. Heterozygous *β* ^*654*^ mice exhibit a moderate thalassaemia phenotype characterised by anaemia, splenomegaly and iron deposition, whereas homozygotes are embryonically lethal [31]. This model is widely used to evaluate HSCT–based therapeutic strategies.

For myeloablation in WT recipients, 6–8‐week‐old mice were divided into two groups. (1) Normal treatment group: mice received intraperitoneal injections of busulfan for 3 consecutive days, followed by cyclophosphamide for 2 days (BuCy); and (2) FG‐4592 treatment group: mice received intraperitoneal FG‐4592 once daily for 5 days, administered concurrently with the BuCy regimen.

For thalassaemia studies, 6–8‐week‐old *β* ^*654*^ mice were conditioned according to the NF‐08‐TM protocol [32]. (1) Normal treated *β* ^*654*^ group: cyclophosphamide for 2 days, followed by busulfan for 3 days; and (2) FG‐4592 treated *β* ^*654*^ group: FG‐4592 (20 mg/kg) administered intraperitoneally for 5 days concurrently with busulfan (10 mg/kg) and cyclophosphamide (200 mg/kg).

Recipient survival following myeloablation was monitored daily. All animal experiments were reviewed and approved by the Laboratory Animal Management and Ethics Committee of Shanghai Children's Hospital (LLSC2016019) and conducted in accordance with institutional and national guidelines.

### 2.2 Donor Cell Transplantation

CD45.1^+^ donor mice were obtained from the Shanghai Nanfang Research Center for Biomodel Organisms. Bone marrow mononuclear cells (BM‐MNCs) were isolated from 6 to 8‐week‐old CD45.1^+^ donors and filtered through a sterile cell strainer to remove clumps. Chimeric mice were prepared by transplanting allogeneic 45.1^+^ donor cells into the 45.2^+^ recipient mice [33].

Mononuclear cells were separated using Ficoll (Mouse Bone Marrow Mononuclear Cell Isolation Kit, TBD2013CM, Tianjin, China). For transplantation, either 4 × 10^8^ cells/kg or 0.8 × 10^8^ cells/kg were administered via intra‐bone marrow injection (IBMI). Chimerism was assessed by flow cytometry using CD45.1/CD45.2.

Note that IBMI has several advantages over intravenous (IV) delivery: (1) It reduces loss of haematopoietic stem cells (HSCs) in circulation and enhances early engraftment; (2) It places donor cells directly into the marrow microenvironment, potentially reducing GVHD risk; (3) It preserves niche integrity by avoiding dilution in peripheral blood [34, 35].

### 2.3 BM‐MNC Cytological Analysis in Recipient Mice

Lineage‐positive (Lin^+^) and lineage‐negative (Lin^−^) BM‐MNCs were isolated using a magnetic cell sorting system (MACS; Miltenyi Biotec, 130‐090‐858): (1) Haematopoietic stem/progenitor cells were evaluated using Sca‐1 (BD Biosciences 557405) and c‐Kit (BD Biosciences 553356) antibodies. (2) Erythroid cells were labelled using Ter119 (Invitrogen 12‐5921‐82).

HIF‐1α expression was detected by flow cytometry using PE‐conjugated anti–HIF‐1α antibody generated with a Lightning‐Link conjugation kit (Abcam ab102918; antibody NB100‐134, Novus Biologicals).

Apoptosis assays were performed using Annexin V–based kits (Beyotime C1062, C1077), and cell‐cycle analysis was performed using Beyotime C1052. In vitro, Lin^−^Sca‐1^+^ and Lin^−^Sca‐1^−^ cells were treated with LW6 (HIF‐1α inhibitor; TargetMol T3494) or DMOG (HIF‐PH inhibitor; TargetMol T1939) during Bu or Cy exposure for 24 h.

Donor chimerism was quantified using PE anti–CD45.1 (BD Pharmingen 553776) and APC anti–CD45.2 (BD Pharmingen 558702).

### 2.4 Haematological and Histological Analyses

Peripheral blood was collected regularly for complete blood cell (CBC) counts and Wright–Giemsa staining (BASO BA4017). Bone marrow smears were also Wright–Giemsa–stained.

At 24 weeks post‐transplantation, liver, spleen and other organs were harvested for histological evaluation, including: Haematoxylin–eosin staining (BASO BA4025) and Prussian blue staining for iron deposition (BASO BA4089B).

### 2.5 Phospho‐Specific Protein Microarray Analysis

A phospho‐specific antibody microarray (CSP100_Plus, Full Moon BioSystems, USA) containing 304 antibodies targeting 157 phospho‐sites and 144 corresponding nonphosphorylated proteins was used to identify signalling pathways altered by FG‐4592 during myeloablation.

Arrays were processed by Wayen Biotechnology (Shanghai, China) per manufacturer instructions. Data acquisition was performed with a SureScan Dx Microarray Scanner, and phosphorylation ratios were calculated as follows: phosphorylation ratio = phospho‐signal/unphospho‐signal, with ≥ 1.2‐fold considered significant.

Enrichment analyses were conducted using WebGestalt (https://www.webgestalt.org), including Mammalian Phenotype, WikiPathway and GO terms.

Validation of signalling molecules was performed by Western blotting using the following antibodies: c‐RAF (Affinity AF6065), phospho‐c‐RAF (Ser259) (AF3064); MEK1/2 (Affinity AF6385), phospho‐MEK1/2 (Ser217/218) (AF3384); p44/42 MAPK (Beyotime AG2954), phospho‐p44/42 MAPK (Thr202/Tyr204) (Beyotime AB3011); p53 (Affinity AF0879), phospho‐p53 (Ser315) (Sabbiotech 11100); BAX (Affinity AF0120), phospho‐Bax (Thr167) (ImmunoWay YP1169).

## 3 Results

### 3.1 FG‐4592 Enhances Donor‐Cell Transplantation Efficiency in Wild‐Type Recipients

Roxadustat (FG‐4592) has been reported to influence erythropoiesis, iron metabolism and cellular stress responses, suggesting that it may modulate the bone marrow milieu during myeloablation. To determine whether FG‐4592 improves haematopoietic reconstruction in vivo, WT recipient mice were conditioned with BuCy and transplanted with donor CD45.1^+^ BM‐MNCs.

To first assess whether FG‐4592 supports engraftment under conditions of saturated donor‐cell availability, WT recipients were transplanted with 4 × 10^8^/kg CD45.1^+^ BM‐MNCs, resulting in chimerism exceeding 95% in both normally treated and FG‐4592–treated groups (Figure S1A). RBC, WBC and HCT values were comparable across groups (Figure S1B). These observations are consistent with Ashihara et al., who demonstrated that infusion of ≥ 5 × 10^6^/kg CD34^+^ cells accelerates haematopoietic recovery clinically [36]. Thus, under conditions of abundant donor input, FG‐4592 does not alter overall chimerism.

To more sensitively evaluate the effect of FG‐4592 on donor‐cell engraftment, we reduced the transplanted donor‐cell dose to 0.8 × 10^8^/kg CD45.1^+^ BM‐MNCs. On Day 2 post‐transplantation, RBC counts did not differ significantly between groups; however, WBC levels were significantly lower in FG‐4592–treated mice (*p* < 0.05), with a more striking reduction in lymphocyte counts (*p* < 0.01). Cell‐cycle analysis revealed that the number of Lin^−^ cells in the G0/G1 phase was markedly reduced in both conditioned groups relative to WT controls (*p* < 0.05), whereas FG‐4592 did not significantly alter the cycling state of Lin^−^Sca‐1^+^ cells (Figure S2A–C). These findings suggest that FG‐4592 transiently suppresses Lin^−^ cell expansion without impairing haematopoietic stem/progenitor cycling.

In contrast, Lin^+^ cells exhibited a significantly higher proportion in G0/G1 and a lower proportion in G2/S/M in FG‐4592–treated mice compared with normally treated controls (*p* < 0.01; Figure S2D–E), indicating reduced proliferation of non‐HSC haematopoietic cells. This reduction parallels the early decline in lymphocytes and supports improved donor‐cell access to niche space.

**FIGURE 1:**
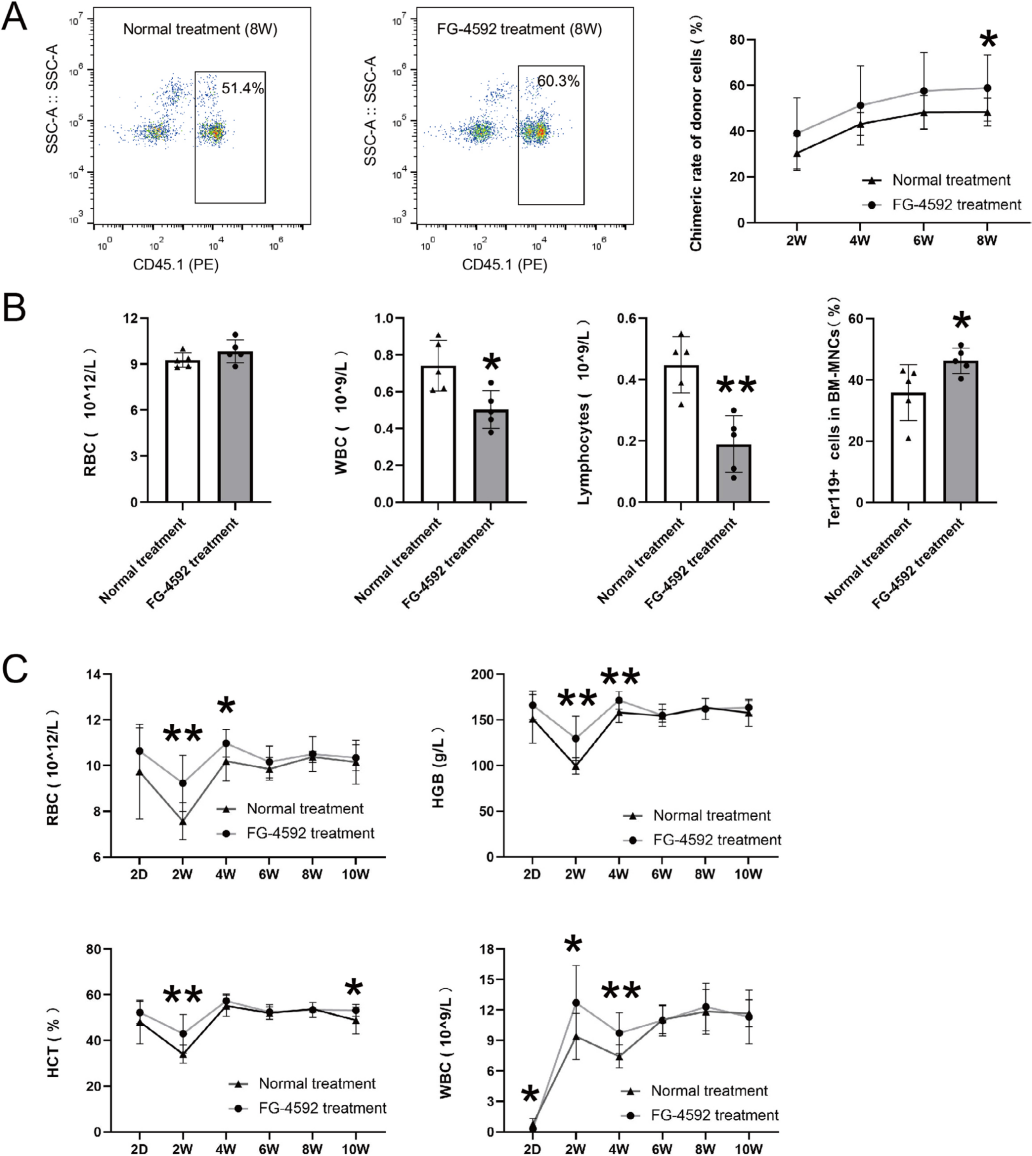
Donor‐cell chimerism and peripheral blood parameters in wild‐type recipients after transplantation. (A) Flow cytometry analysis of donor‐derived CD45.1^+^ cells in the peripheral blood of recipient mice (*n* = 10). (B) Comparison of peripheral blood parameters between normally treated and FG‐4592–treated recipient mice on Day 2 after transplantation with low‐dose BM‐MNCs (*n* = 5 per group). (C) Kinetics of erythrocyte, leukocyte, haemoglobin (HGB) and haematocrit (HCT) levels following low‐dose BM‐MNC transplantation in normally treated versus FG‐4592–treated groups (*n* = 10 per group). *p < 0.05; **p < 0.01.

Consistent with these early differences, donor‐cell chimerism in FG‐4592–treated recipients progressively increased and stabilised beyond Week 8 (Figure 1A). Chimerism was approximately 15% higher in FG‐4592–treated mice than in normally treated mice at steady state, demonstrating that FG‐4592 enhances donor‐cell implantation efficiency and reduces the number of donor cells required to achieve comparable engraftment.

#### 3.1.1 Kinetics of Key Blood Parameters After Transplantation

FG‐4592 administration resulted in a distinct haematologic pattern during early recovery. On average, FG‐4592–treated mice exhibited higher RBC counts but lower WBC counts, suggesting an altered marrow microenvironment conducive to erythroid recovery.

Following transplantation, RBC, HGB and HCT levels initially declined, whereas WBC counts rose during the first 2 weeks. Between weeks 2 and 4, RBC, HGB and HCT increased steadily while WBC counts decreased (Figure 1B). The proportion of Ter119^+^ erythroid cells within BM‐MNCs was significantly higher in FG‐4592–treated mice than in normally treated controls (*p* < 0.05), indicating enhanced erythroid differentiation or recovery.

By Week 6, all major hematologic parameters approached stability. At Week 10, RBC, HGB and WBC levels did not differ between recipient groups and WT controls, although HCT remained modestly altered. Platelet counts were lower in FG‐4592–treated mice than in untreated WT controls (*p* < 0.05), but this reduction was not statistically significant relative to normally treated recipients (Figure 1C; Table S1).

Together, these findings demonstrate that FG‐4592 is well tolerated and enhances donor‐cell engraftment dynamics, as reflected by higher donor chimerism and favourable erythroid recovery.

### 3.2 FG‐4592–Treated Myeloablation Improves Anaemia and Haematopoietic Reconstruction in *β* ^*654*^ Thalassaemia Mice

HSCT is a potential curative approach for severe thalassaemia, but limited HSC availability often constrains clinical outcomes. Strategies that increase donor‐cell engraftment efficiency while reducing required donor doses are therefore desirable. We next evaluated whether FG‐4592 enhances transplantation efficacy in *β* ^*654*^ thalassaemia mice using a reduced donor‐cell dose.

**FIGURE 2:**
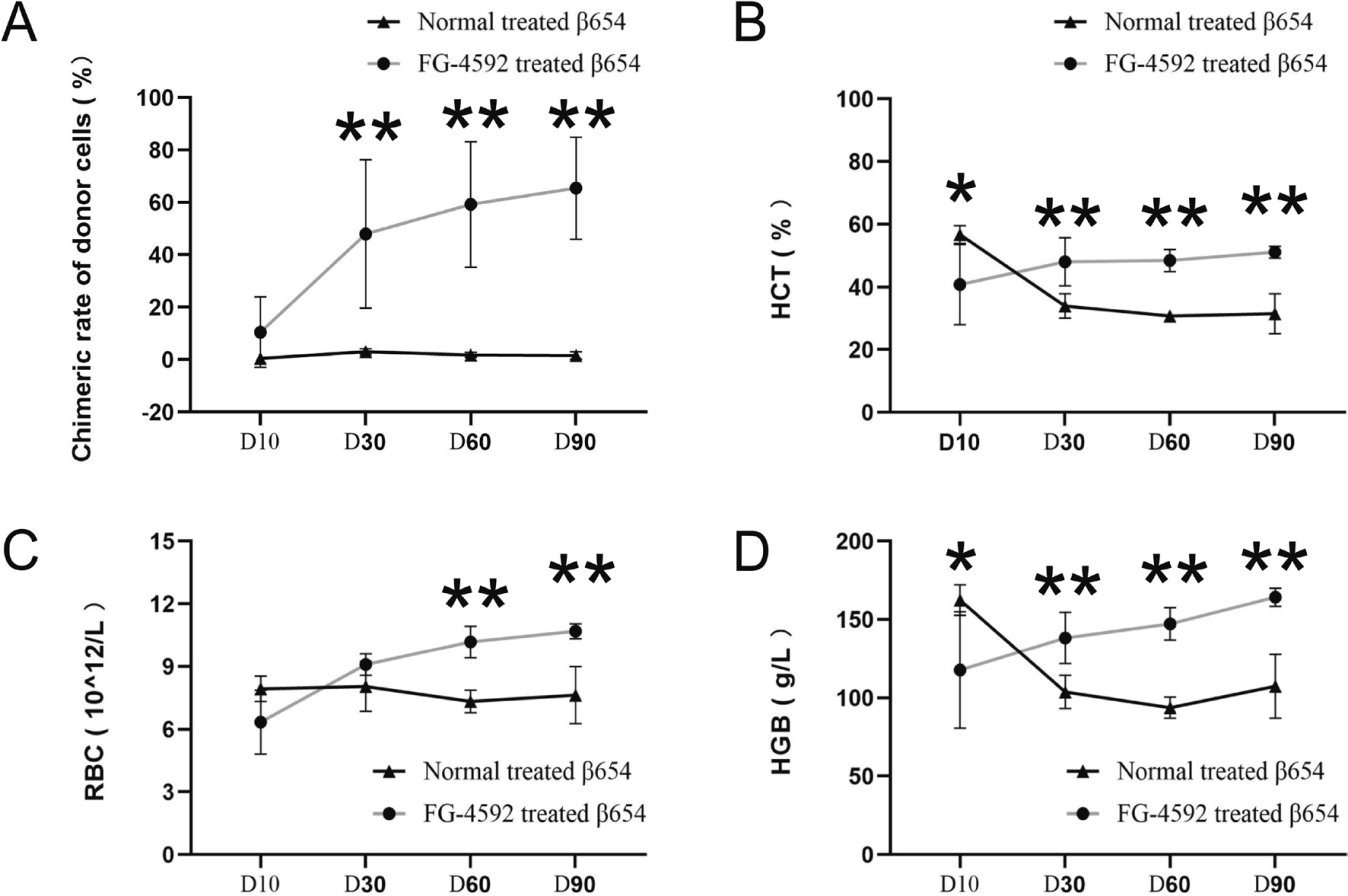
Donor‐cell chimerism and blood parameters in *β* ^*654*^ recipient mice after transplantation. (A) Percentage of donor‐derived CD45.1^+^ cells in the peripheral blood of *β* ^*654*^ recipients. (B–D) Routine blood parameters, including HCT, RBC count and HGB concentration, in FG‐4592–treated versus normally treated *β* ^*654*^ recipient mice (*n* = 5 per group). *p < 0.05; **p < 0.01.

Using 0.8 × 10^8^/kg BM‐MNCs, chimerism remained low (approximately 1.5%) and stable in normally treated *β* ^*654*^ recipients. In contrast, FG‐4592–treated *β* ^*654*^ recipients showed steadily increasing donor‐cell chimerism, reaching approximately 65% by Day 90 (*p* < 0.01; Figure 2A). These results indicate that FG‐4592 dramatically improves donor‐cell engraftment in the *β* ^*654*^ model.

Consistent with improved engraftment, FG‐4592–treated *β* ^*654*^ mice displayed significantly higher HCT, RBC and HGB levels compared with normally treated *β* ^*654*^ mice, whose values declined during the 60 days following transplantation. By Day 90, hematologic parameters were markedly improved in FG‐4592–treated recipients (*p* < 0.01; Figure 2B–D).

**FIGURE 3:**
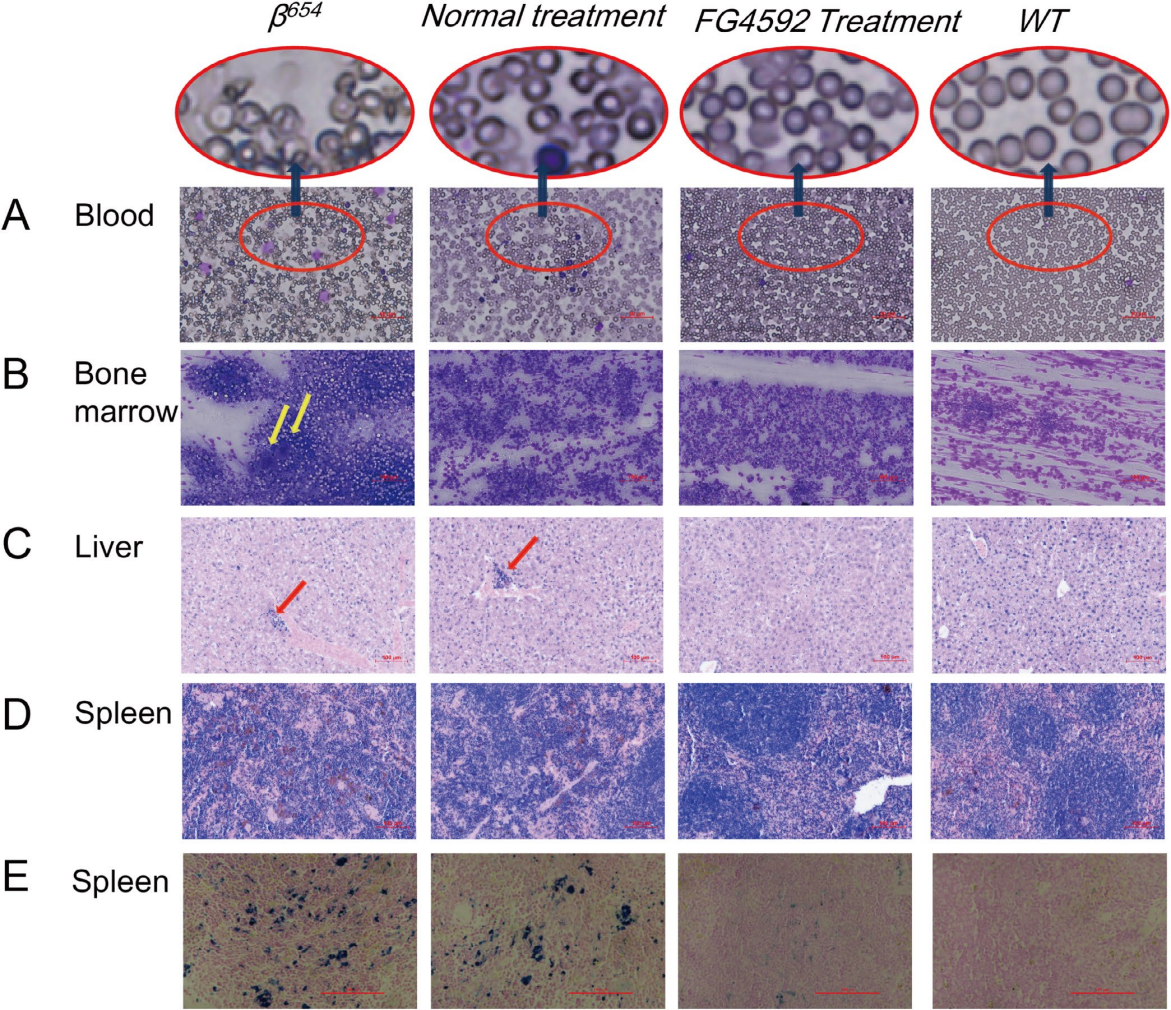
Morphology of blood cells and tissue histology in *β* ^*654*^ recipients after transplantation. (A) Wright–Giemsa–stained peripheral blood smears (400×). (B) Wright–Giemsa–stained bone marrow smears (400×); yellow arrows indicate megakaryocytes. (C) Haematoxylin–eosin–stained liver sections (400×); red arrows indicate erythroid precursors. (D) Hematoxylineosin–stained spleen sections (400×). (E) Ferrocyanide iron staining of spleen samples (400×).

Microscopic examination further supported these improvements. Blood smears and bone marrow cytology revealed that RBCs from FG‐4592–treated *β* ^*654*^ mice displayed near‐normal morphology with fewer microcytic and hypochromic features (Figure 3A). Atypical and fragmented cells were significantly reduced. In contrast, normally treated *β* ^*654*^ mice retained classical thalassaemia cellular phenotypes, including elevated WBC counts.

Bone marrow smears from normally treated *β* ^*654*^ mice contained more cells with a high nuclear‐to‐cytoplasmic ratio, whereas FG‐4592–treated mice exhibited more normalised marrow morphology (Figure 3B). Secondary transplantation using BM‐MNCs from FG‐4592–treated *β* ^*654*^ mice did not transmit thalassemic RBC phenotypes, whereas donor cells from normally treated *β* ^*654*^ mice retained such abnormalities (Figure S3). These results indicate superior haematopoietic correction achieved in FG‐4592–treated recipients.

Histopathological analyses offered additional evidence of phenotypic improvement. In the livers of normally treated *β* ^*654*^ mice, immature erythroid cells were frequently observed in sinusoids, resembling untreated *β* ^*654*^ pathology. These aberrant cells were largely absent in FG‐4592–treated mice (Figure 3C). In the spleen, *β* ^*654*^ pathology typically includes a blurred boundary between red and white pulp. FG‐4592–treated mice exhibited near‐normal splenic architecture, with a clear demarcation and reduced RBC deposition (Figure 3D).

Iron staining analyses (Figure 3E; Figure S4) further revealed that FG‐4592–treated *β* ^*654*^ mice accumulated less hemosiderin in the spleen, consistent with amelioration of iron overload.

Together, these findings demonstrate that FG‐4592 substantially enhances donor‐cell engraftment, improves erythropoiesis, corrects thalassemic blood phenotypes and mitigates pathological organ changes in *β* ^*654*^ mice following HSCT.

### 3.3 FG‐4592–Treated Myeloablation Enhances Recipient Survival and Optimises the Niche for Donor‐Cell Implantation

Successful HSCT depends heavily on the effectiveness of myeloablation. Efficient clearance of recipient HSCs, lymphocytes and other self‐renewing cell populations reduces both host‐versus‐graft and graft‐versus‐host responses, while an appropriate haematopoietic microenvironment with adequate RBCs is essential for maintaining physiological stability during this vulnerable period. Because FG‐4592 enhances cellular stress responses and modulates erythropoiesis, we asked whether its co‐administration during BuCy conditioning would improve niche quality and survival outcomes.

Flow cytometry analysis revealed that FG‐4592 significantly increased HIF‐1α protein levels in Lin^+^ cells relative to normally treated controls (*p* < 0.05), while Lin^−^ cells showed no significant difference (Figure S5A). These results indicate that FG‐4592 preferentially stabilises HIF‐1α in non‐HSC haematopoietic lineages during conditioning, potentially enhancing their resilience to chemotoxic stress and protecting the marrow environment.

As myeloablation intensity increased, mouse mortality rose accordingly. However, the slope of mortality was markedly attenuated in FG‐4592–treated recipients (Figure S5B). Under a standard BuCy regimen (busulfan 10 mg/kg + cyclophosphamide 200 mg/kg), 90% of normally treated mice died within 90 days, whereas mortality was only 10% in FG‐4592–treated mice. These findings suggest that FG‐4592 helps maintain a healthier marrow milieu during myeloablation, thereby improving overall survival.

**FIGURE 4:**
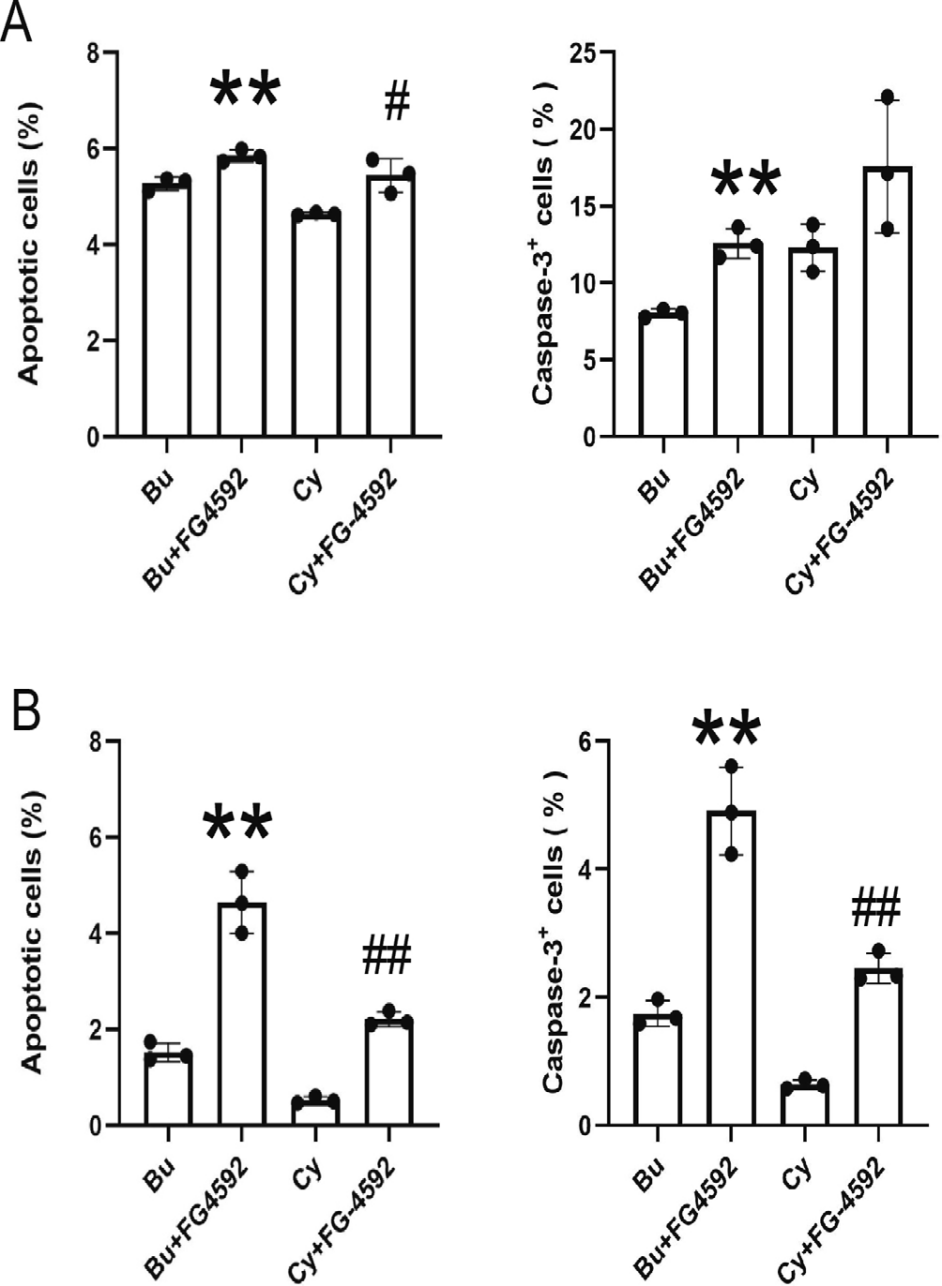
Apoptosis of recipient haematopoietic and peripheral blood cells following BuCy conditioning with or without FG‐4592. (A) Detection of apoptotic Lin^−^c‐Kit^+^ cells in bone marrow after treatment. (B) Apoptosis of peripheral white blood cells 24 h after intraperitoneal injection of busulfan, busulfan with FG‐4592, cyclophosphamide, or cyclophosphamide with FG‐4592 (*n* = 3). Apoptosis was assessed using Annexin V‐mCherry, and expression of the apoptotic effector protein Caspase‐3 was also measured. Statistical comparisons: busulfan + FG‐4592 versus busulfan: Statistical analysis: FG‐4592 treatment vs. Normal treatment: **p < 0.01; cyclophosphamide + FG‐4592 versus cyclophosphamide: ^#^ *p* < 0.05, ^##^ *p* < 0.01.

We next evaluated the apoptotic response induced by busulfan or cyclophosphamide with or without FG‐4592. Both combinations increased apoptosis in Lin^−^c‐Kit^+^ haematopoietic progenitors and in circulating white blood cells (Figure 4). In vitro experiments further showed that FG‐4592 enhanced busulfan and cyclophosphamide induced apoptosis in both Lin^−^Sca‐1^+^ haematopoietic progenitors and Lin^−^Sca‐1^−^ stromal‐like cells (Figure S6). These results support the conclusion that FG‐4592 deepens recipient‐cell clearance during myeloablation.

**FIGURE 5:**
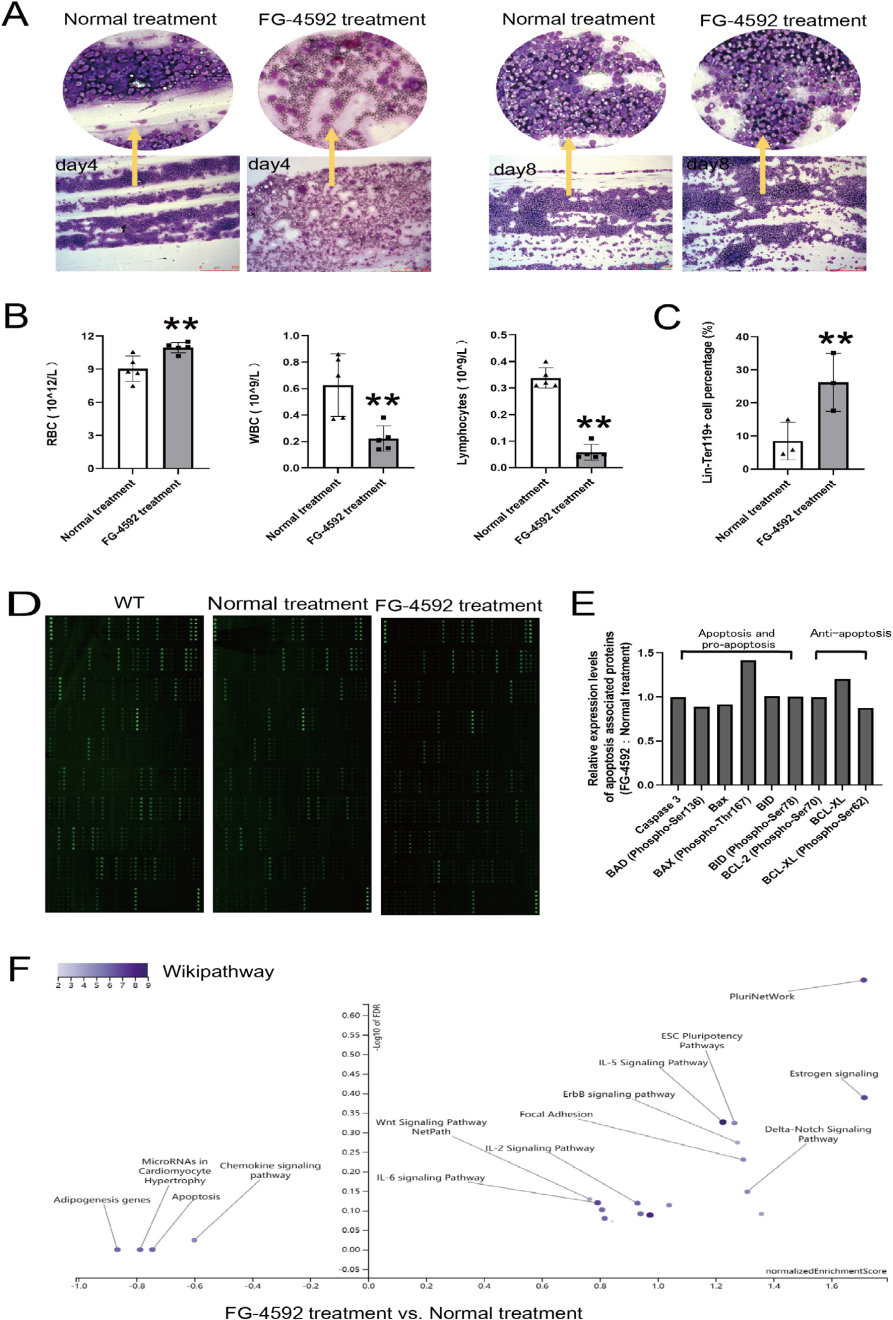
Phenotypic changes in bone marrow and peripheral blood after myeloablation with or without FG‐4592. (A) HE‐stained bone marrow smears. (B) Comparison of RBC, WBC and lymphocyte counts between normally treated and FG‐4592–treated mice 48 h after myeloablation (*n* = 5). (C) Ratio of Ter119^+^ erythroid cells in bone marrow smears 48 h after myeloablation (normal‐treated group: *n* = 3; FG‐4592–treated group: *n* = 3). Statistical analysis: FG‐4592 treatment vs. Normal treatment: **p < 0.01. (D) Phospho‐specific protein microarray profiling of BM‐MNCs: Wild‐type, normally treated and FG‐4592–treated groups. (E) Relative expression levels of apoptosis‐related proteins in FG‐4592– versus normally treated groups. (F) WikiPathway analysis of upregulated phosphorylated proteins in FG‐4592–treated versus normally treated BM‐MNCs.

Bone marrow cytology provided additional insight. Wright–Giemsa staining on Day 4 of conditioning revealed higher numbers of RBCs in FG‐4592–treated mice. By Day 8, these RBCs were progressively replaced by nucleated donor‐derived cells, a pattern not observed in normally treated mice (Figure 5A). In parallel, WBC counts including lymphocytes were significantly lower in the FG‐4592–treated group (Figure 5B, *p* < 0.01), reflecting enhanced depletion of residual recipient immune cells. This sequence preservation of essential RBCs while efficiently eliminating leukocytes supports the physiological stability of recipients while facilitating donor‐cell engraftment.

Previous clinical observations by Besarab et al. showed that a single 1.0 mg/kg dose of FG‐4592 induces a transient erythropoietin (EPO) surge, peaking at 10 h and returning to baseline within 24–48 h [37]. In our model, this transient EPO effect likely contributes to short‐term RBC support. In normally treated mice, in contrast, erythroid recovery was insufficient, potentially compromising tolerance to myeloablation.

Flow cytometry of erythroid‐marked Ter119^+^ cells in the Lin^−^ compartment revealed a significant increase in erythroid cells in FG‐4592–treated mice compared with controls (Figure 5C). These findings indicate that FG‐4592 enhances erythroid expansion during early recovery, maintaining RBC levels while facilitating clearance of HSCs and inflammatory cells.

Collectively, these results demonstrate that FG‐4592 supports a more efficient and physiologically balanced myeloablative process—reducing mortality, improving niche quality, deepening recipient‐cell depletion and promoting donor‐cell implantation.

### 3.4 FG‐4592 Modulates Apoptotic Fate Determination in Recipient Bone Marrow Cells Revealed by Phospho‐Specific Protein Microarray

To elucidate the molecular mechanisms underlying FG‐4592–enhanced myeloablation, we performed a phospho‐specific protein microarray on BM‐MNCs collected after conditioning. The fluorescence signals for phosphorylated proteins are shown in Figure 5D. Most phosphorylation events were elevated in both normally treated and FG‐4592–treated groups compared to WT, whereas certain proteins—including phosphorylated Rb and Cyclin E1—were reduced (Figure S4A), consistent with diminished cell proliferation during myeloablative stress.

Apoptosis‐related signalling differed notably among the three groups (S7–S9). FG‐4592 enhanced apoptosis primarily through activation of the MAPK and p53 signalling pathways. In the MAPK cascade, FG‐4592 increased phosphorylation of RAF1, leading to activation of MEK1 (MAP2K1) and downstream ERK1/2 (MAPK1/3). Activated ERK1/2 is known to promote apoptosis under stress conditions, providing a mechanistic explanation for the enhanced cell death observed during conditioning.

The p53 pathway was also strongly modulated. Increased phosphorylation of p53 (Phospho‐Ser315) activated the downstream apoptotic effector BAX (Phospho‐Thr167), promoting programmed cell death in target populations (Figure S10A). Western blot analyses confirmed that the p53 → BAX pathway was preferentially activated in Lin^−^Sca‐1^+^ HSC/progenitor cells. In contrast, the RAF1 → MEK1 → MAPK pathway was more strongly induced in Lin^−^Sca‐1^−^ stromal‐like cells, though active in both populations (Figure S10B).

The effects of FG‐4592 during busulfan and cyclophosphamide treatment were examined in vitro, in the presence of LW6 (a HIF‐1α inhibitor) or DMOG (a HIF prolyl hydroxylase inhibitor). In non‐haematopoietic Lin^−^Sca‐1^−^ and Lin^−^ cells, both HIF‐PH inhibition and HIF‐1α stabilisation were associated with reduced apoptosis. In contrast, in Lin^−^Sca‐1^+^ haematopoietic progenitor cells, inhibition of HIF‐PH increased apoptosis. Combined FG‐4592 and DMOG treatment further enhanced apoptosis in Lin^−^Sca‐1^+^ cells during myeloablation, whereas cyclophosphamide exerted a more pronounced pro‐apoptotic effect on non‐haematopoietic cells (Figure S11). These results indicate that FG‐4592 regulates apoptotic responses in a cell‐type–dependent manner during myeloablative treatment.

Compared with normally treated mice, FG‐4592–treated recipients showed higher BAX (Phospho‐Thr167)/BAX ratios and lower BCL‐XL (Phospho‐Ser62)/BCL‐XL ratios (Figure 5E), consistent with heightened apoptotic signalling. These changes correspond directly to the enhanced marrow clearance observed in Figure 4.

Pathway enrichment via WikiPathways further indicated that FG‐4592 upregulated EPO receptor signalling and p53‐dependent tissue‐repair pathways. These pathways may contribute to the maintenance of RBC homeostasis and regulation of stress responses during myeloablation. In contrast, normally treated mice exhibited increased activation of FAS signalling, heat‐shock stress pathways and MAPK‐driven stress responses (Figure S4B), which may exacerbate tissue damage.

Moreover, FG‐4592–treated mice displayed enhanced activation of stem‐cell survival pathways such as PluriNetWork (Figure 5F), suggesting an improved marrow microenvironment for donor‐cell implantation. MP analysis indicated that the phosphorylated proteins upregulated in both treatment groups relative to WT were primarily enriched in processes related to protein phosphorylation, apoptosis and cytokine responses; however, the normally treated group showed additional phenotypes such as reduced B‐cell numbers and abnormal cell‐cycle dynamics (Figure S4C).

Together, these findings indicate that FG‐4592 modulates the apoptotic landscape of conditioned marrow, promoting efficient clearance of recipient cells while simultaneously enhancing environments that support RBC stability, reduce inflammation and maintain stem‐cell potential—all contributing to improved transplantation outcomes.

## 4 Discussion

Myeloablation is a critical determinant of successful HSCT. Adequate clearance of recipient haematopoietic and immune cells reduces competition within the marrow niche and enables efficient engraftment of donor HSCs. However, current chemotherapy‐based regimens, particularly BuCy, impose significant hematologic and systemic toxicity [5, 6, 7, 8, 9, 10, 11, 12, 37, 38, 39], limiting their tolerability and occasionally compromising transplantation outcomes. This study identifies roxadustat (FG‐4592), a HIF‐prolyl hydroxylase inhibitor [13], as a promising adjunct that enhances the depth and quality of myeloablation while simultaneously supporting physiological stability and donor‐cell engraftment.

Using both WT and *β* ^*654*^ thalassaemia mouse models, we found that FG‐4592 co‐administration during myeloablation significantly improved donor‐cell chimerism, even when donor BM‐MNC doses were reduced. In WT recipients, FG‐4592 enhanced early marrow clearance, as evidenced by reduced WBC and lymphocyte counts immediately after transplantation and facilitated efficient donor‐cell residency, leading to higher long‐term chimerism. In *β* ^*654*^ mice, FG‐4592 dramatically improved chimeric levels—approximately 65% by Day 90 compared with only 1.5% in normally treated recipients—demonstrating its potential to improve engraftment outcomes in settings of ineffective erythropoiesis or limited donor‐cell availability.

FG‐4592 also enhanced early erythroid stability and post‐transplant RBC recovery. Roxadustat is known to promote erythropoiesis through HIF‐1α stabilisation, EPO induction and optimisation of iron metabolism [16, 17, 18, 19], mechanisms consistent with our observation of higher Ter119^+^ erythroid proportions in FG‐4592–treated mice. In *β* ^*654*^ thalassaemia mice, FG‐4592 normalised RBC morphology, corrected microcytic hypochromic features and reduced abnormal blood cell forms. These hematologic improvements were accompanied by tissue‐level benefits: FG‐4592–treated *β* ^*654*^ mice exhibited restored liver and spleen architecture, reduced extramedullary haematopoiesis, and markedly lower hemosiderin deposition. These findings complement previous evidence that roxadustat supports tissue protection and reduces inflammatory injury in multiple organs, including the kidney, heart, colon and lung [14, 15, 24, 25, 26, 27, 28, 29].

One of the most striking observations was the substantial improvement in survival among FG‐4592–treated mice. Under standard BuCy conditioning, mortality increased sharply, consistent with known BuCy toxicities [7, 8, 9, 10, 11, 12], reaching 90% in normally treated recipients. In contrast, only 10% mortality occurred in FG‐4592–treated mice. This survival benefit likely reflects FG‐4592's ability to stabilise HIF‐1α and enhances cellular resistance to oxidative and cytotoxic stress [13, 14, 15, 24, 25, 26, 27, 28, 29]. Our flow cytometry data showed increased HIF‐1α in Lin^+^ cells, suggesting that FG‐4592 selectively supports non‐HSC haematopoietic populations during cytotoxic injury, thereby maintaining a healthier marrow microenvironment.

Mechanistically, phospho‐specific protein microarray analysis revealed that FG‐4592 enhanced activation of apoptotic signalling pathways relevant to conditioning. The RAF1 → MEK1 → MAPK cascade and the p53 → BAX pathway were both activated in FG‐4592–treated mice, consistent with increased apoptosis in both haematopoietic progenitors and stromal‐like cells. These findings align with prior evidence that HIF pathway modulation can influence cellular stress responses and apoptotic sensitivity [13, 24, 25, 26]. At the same time, FG‐4592 upregulated pathways associated with erythropoietin receptor signalling and tissue repair, consistent with known erythropoietic and cytoprotective effects of roxadustat [16, 17, 18, 19, 20, 21, 22, 23]. This dual action—enhanced apoptotic clearance coupled with improved erythroid support—provides a mechanistic basis for the superior engraftment and reduced toxicity observed in vivo.

A noteworthy feature of FG‐4592 treatment was the balanced haematologic response. While FG‐4592 promoted deeper depletion of WBCs and lymphocytes—facilitating donor‐cell niche access—it simultaneously maintained RBC levels, supporting oxygen delivery during conditioning. This behaviour is consistent with the transient EPO elevation observed clinically after roxadustat administration [40] and may account for improved tolerance to myeloablation.

Taken together, our findings demonstrate that FG‐4592 enhances conditioning efficiency through several coordinated mechanisms: (1) deepened apoptosis of recipient haematopoietic and stromal cells; (2) improved marrow niche accessibility for donor HSCs; (3) supported erythroid recovery and RBC homeostasis; (4) reduced conditioning‐associated tissue injury and inflammatory stress; and (5) markedly improved survival outcomes. These benefits were observed in both WT and *β* ^*654*^ thalassaemia models, indicating broad therapeutic potential.

To our knowledge, this is the first study to show that a HIF‐prolyl hydroxylase inhibitor can be co‐administered during myeloablation to enhance donor‐cell engraftment while mitigating the adverse effects of cytotoxic conditioning. Given the clinical availability and established safety profile of roxadustat [20, 21, 22, 23], these findings support further investigation of its use as an adjunct to standard conditioning regimens. Future studies should explore optimal dosing strategies, timing and potential clinical applications in patients with limited donor‐cell availability or underlying ineffective erythropoiesis.

## 5 Conclusion

This study demonstrates that co‐administration of roxadustat (FG‐4592) during BuCy conditioning enhances donor‐cell engraftment, improves haematopoietic reconstruction and reduces treatment‐related toxicity in both WT and *β* ^*654*^ thalassaemia mice. Roxadustat deepened early marrow clearance, supported erythroid stability and markedly improved recipient survival, while activating apoptotic and erythropoietic pathways that optimise the marrow niche for donor HSC implantation.

These findings indicate that FG‐4592 can enhance the effectiveness of conventional myeloablation and improve transplantation outcomes without increasing toxicity. Given its established clinical use and safety profile, roxadustat represents a promising adjunct to standard conditioning regimens and warrants further evaluation in haematopoietic stem cell transplantation.

## Author Contributions

Guanheng Yang, Jingzhi Zhang and Fanyi Zeng conceived and designed the project. Guanheng Yang, Jiahui Wu, Xinbing Guo, Wenchen Shen, Xiao Sang and Xiuli Gong performed the characterisation studies, experiments and biological analyses. Guanheng Yang, Jingzhi Zhang and Fanyi Zeng contributed to manuscript writing, review and editing. Shu‐Zhen Huang, Jingzhi Zhang and Fanyi Zeng supervised the study and guided project development. All authors contributed to data interpretation and approved the final manuscript.

## Funding

This work was supported by the National Key Research and Development Program of China (2024YFC2707002, 2019YFA0801402), National Natural Science Foundation of China (U23A20498, 82271890, 32570951), Innovative Research Team of High‐level Local University in Shanghai (SHSMU‐ZDCX20212200), the Macau Science and Technology Development Fund (FDCT) (0092/2022/A2, 003/2022/ALC).

## Conflicts of Interest

The authors declare no conflicts of interest.

## Supporting information


**Figure S1:** Chimerism and peripheral blood parameters in WT recipients transplanted with high‐dose BM‐MNCs. (A) CD45.1^+^ cell chimerism in peripheral blood. Chimerism of the donor cells in the peripheral blood cells revealed that high‐dose BM‐MNCs transplantation could ensure effective replacement of recipient haematopoietic cells by donor cells: (B) RBC, WBC and HCT comparisons across different groups. Selected parameters of blood routine test of the recipient mice, including WBC, RBC and HCT, 4 weeks after HSCT, revealed that high‐dose donor cells had almost the same effect between the normal treated group and the FG‐4592 treated group. WT *n* = 5, Normal treatment *n* = 3, FG‐4592 treatment *n* = 3. The number of haematopoietic stem cell transplantations was 4 × 10^8^/kg.
**Figure S2:** Cell‐cycle analysis of Lin^−^ and Lin^+^ BM‐MNCs on Day 2 post‐transplantation. Comparison of the ratios at the same cell cycle states in bone marrow cells on the second day after low‐dose BM‐MNC transplantation revealed that the slower growth of blood cells in non‐haematopoietic cells occurred in the FG‐4592‐treated group. (A) Flow cytometry detection of the cell cycle of Lin^−^ cells. (B) Flow cytometry detection of the cell cycle of Lin^+^ cells. (C) Cell‐cycle analysis of Lin^−^ cells between treatments. (D) Cell‐cycle analysis of Lin^−^Sca‐1^+^ cells between treatments. (E) Cell‐cycle analysis of *Lin* ^*+*^ cells between treatments. White bar: G0/G1 cells. Black bar: G2/S/M cells. WT group (*n* = 5), Normal treatment group (*n* = 5), FG‐4592 treatment group (*n* = 5). Normal treated group compared with that of WT group, ^▲^ *p* < 0.01, ^▲▲^ *p* < 0.05, FG‐4592 treated group compared with that of Normal treated group: *p < 0.05, **p < 0.01; FG‐4592 treated group compared with that of WT: ^#^ *p* < 0.05, ^##^ *p* < 0.01. Compared to WT mice, the resting phase characteristics of haematopoietic cells in all myeloablated mice was gradually lost; Compared to normal myeloablated mice, FG‐4592 treatment helps to stabilise the resting phase of haematopoietic cells, but inhibits their expansion.
**Figure S3:** Secondary transplantation results and erythroid morphology showing corrected RBC phenotypes from FG‐4592–treated *β* ^*654*^ donor marrow.
**Figure S4:** The comparison of the phosphorylated protein expression profiles of the BM‐MNCs for those of three groups (FG‐4592‐treated group, Normal‐treated group and WT group). (A) Heat maps of the phosphorylated proteins expressed in the BM‐MNC of those three groups, indicating expression of phosphorylated proteins involved in proliferation and apoptosis. (B, C) Enrichment analysis on those upregulated phosphorylated proteins reveals that the activity and functionality of proteins (The comparison for those of fold‐changes ≥ 1.2). (B) WikiPathways comparison between treatment groups (upregulated phosphorylated proteins of the Normal treated group vs. the WT group, the FG‐4592 treated group vs. the WT group). (C) MP analysis including B‐cell and cell‐cycle phenotypes. MP and GO analysis of those upregulated phosphorylated proteins. As a summary, the outcomes of the above analysis revealed that the strengthening of EPO receptor signalling and PluriNetWork by the administration of FG‐4592, while the wakening of B cell proliferation and T cell proliferation pathways, for creating a better internal environment for haematopoietic cell implantation.
**Figure S5:** HIF‐1α protein was stabilised in vivo upon co‐administration of FG‐4592 during the course of myeloablation, leading to an increase in survival rates of the myeloablated mice. (A) HIF‐1α expression in Lin^+^ and Lin^−^ cells following conditioning. Intracellular HIF‐1α protein levels in the Lin^−^ or Lin^+^ cells were measured by flow cytometry, and a significant increase in expression of HIF‐1α for those FG‐4592‐treated Lin^+^ cells was observed. The recipient mice were divided into two groups: one was treated with busulfan for 3 days, then with cyclophosphamide (BuCy) for another 2 days (Normal treatment group), while the other group was injected with FG‐4592 intraperitoneally daily for 5 consecutive days during BuCy‐based myeloablation. FG‐4592 treatment group versus normal treatment group, *n* = 3, *p < 0.05. (B) The survival curve of the mice after the normal treatment versus the FG‐4592 treatment. A much increase of the survival rate for those of FG‐4592 treated mice was observed. The dosage of busulfan was shown in the Figure 1A. Cyclophosphamide 200 mg/kg, FG‐4592 20 mg/kg, *n* = 10 for each group.
**Figure S6:** In vitro apoptosis of Lin^−^Sca‐1^+^ and Lin^−^Sca‐1^−^ cells following Bu/Cy ± FG‐4592 (Detection of cell apoptosis after treating cells with demyelinating reagents in vitro for 24 h). (A) Apoptosis analysis of Lin^*−*^Sca‐1^*+*^ cells. (B) Apoptosis analysis of Lin^−^Sca‐1^−^ cells. (C) Apoptosis analysis of Lin^+^ cells. Busulfan with FG‐4592 versus busulfan: **p < 0.01 or cyclophosphamide with FG‐4592 versus cyclophosphamide: ^##^ *p < 0.01*. *n* = 3.
**Figure S7:** Pathway map for apoptosis signalling of Normal treatment versus WT.
**Figure S8:** Pathway map for apoptosis signalling of FG‐4592 treatment versus WT.
**Figure S9:** Pathway map for apoptosis signalling of FG‐4592 treatment versus normal treatment.
**Figure S10:** FG‐4592 promotes apoptosis‐related signalling pathways during BuCy myeloablation: Inference and validation. (A) Activation of MAPK and p53 apoptotic pathways. Two signalling pathways were inferred to be involved in FG‐4592‐mediated apoptosis promotion during myeloablation. The path included MAPK signalling and p53 signalling. (B) Western blot validation in Lin^−^Sca‐1^+^, Lin^−^Sca‐1^−^ and BM‐MNCs. Changes in signalling pathways during apoptosis in Lin^−^Sca‐1^+^ haematopoietic cells, Lin^−^Sca‐1^−^ non‐haematopoietic cells, and total BM‐MNCs were evidenced by Western blotting of phosphorated proteins.
**Figure S11:** The effect of HIF‐PH inhibition or stabilisation on the apoptosis of various types of BM‐MNCs during in vitro myeloablation by Bu or Cy. (A) The apoptosis ratio of the Lin^−^Sca‐1^+^ cells. (B) The apoptosis ratio of the Lin^−^Sca‐1^−^ cells. ** versus Bu + FG‐4592, *p < 0.01*; # versus Bu + FG‐4592, *p < 0.05*; ^##^ versus Bu + FG‐4592, *p < 0.01*, *n* = 3.
**Table S1:** The outcomes of blood routine test on Week 10 after low‐dose BM‐MNCs transplantation.

## Data Availability

The data that supports the findings of this study are available in the Supporting Information of this article.
