# Supplementary material for: Optimising Haematopoietic Stem Cell Transplantation: Enhancing Myeloablation Sensitivity and Alleviating Anaemia Using Roxadustat (FG‐4592)
Source: Cell Prolif. 2026 Jul 10;59(11):e70230. doi: 10.1111/cpr.70230 (PMC13352331; doi:10.1111/cpr.70230)
Supplement: Supplementary file 1 [file CPR-59-e70230-s001.docx]

**Optimizing Hematopoietic Stem Cell Transplantation: Enhancing Myeloablation Sensitivity and Alleviating Anemia with Roxadustat (FG-4592)**

**Supplementary Materials**

**Figure S1.** Chimerism and peripheral blood parameters in WT recipients transplanted with high-dose BM-MNCs. **A.** CD45.1⁺ cell chimerism in peripheral blood. Chimerism of the donor cells in the peripheral blood cells revealed that high-dose BM-MNCs transplantation could ensure effective replacement of recipient hematopoietic cells by donor cells. **B.** RBC, WBC, and HCT comparisons across different groups. Selected parameters of blood routine test of the recipient mice, including WBC, RBC, and HCT, 4 weeks after HSCT, revealed that high-dose donor cells had almost the same effect between the normal treated group and the FG-4592 treated group. WT n=5, Normal treatment n=3, FG-4592 treatment n=3. The number of hematopoietic stem cell transplantations was 4×10^8^/kg.

**Figure S2.** Cell-cycle analysis of Lin⁻ and Lin⁺ BM-MNCs on Day 2 post-transplantation. Comparison of the ratios at the same cell cycle states in bone marrow cells on the second day after low-dose BM-MNC transplantation revealed that the slower growth of blood cells in non-hematopoietic cells occurred in the FG-4592-treated group. **A.** Flow cytometry detection of the cell cycle of Lin^-^ cells. **B.** Flow cytometry detection of the cell cycle of Lin^+^ cells. **C.** Cell-cycle analysis of Lin⁻ cells between treatments. **D.** Cell-cycle analysis of Lin⁻Sca-1^+^ cells between treatments. **E.** Cell-cycle analysis of *Lin^+^* cells between treatments. White bar: G0/G1 cells. Black bar: G2/S/M cells.

WT group (n=5), Normal treatment group (n=5), FG-4592 treatment group (n=5). Normal treated group compared with that of WT group, ^▲^*P<0.01*，^▲▲^*P<0.05*； FG-4592 treated group compared with that of Normal treated group: **P<0.05*，***P<0.01*; FG-4592 treated group compared with that of WT: ^#^*P<0.05*，^##^*P<0.01*. Compared to WT mice, the resting phase characteristics of hematopoietic cells in all myeloablated mice was gradually lost; Compared to normal myeloablated mice, FG-4592 treatment helps to stabilize the resting phase of hematopoietic cells, but inhibits their expansion.

**Figure S3.** Secondary transplantation results and erythroid morphology showing corrected RBC phenotypes from FG-4592–treated β⁶⁵⁴ donor marrow.

**Figure S4.** The comparison of the phosphorylated protein expression profiles of the BM-MNCs for those of three groups (FG-4592-treated group, Normal-treated group, and WT group). **A.** Heat maps of the phosphorylated proteins expressed in the BM-MNC of those three groups, indicating expression of phosphorylated proteins involved in proliferation and apoptosis. **B-C.** Enrichment analysis on those upregulated phosphorylated proteins reveals that the activity and functionality of proteins (The comparison for those of fold-changes ≥1.2). **B.** WikiPathways comparison between treatment groups (upregulated phosphorylated proteins of the Normal treated group vs. the WT group, the FG-4592 treated group vs. the WT group). **C.** MP analysis including B-cell and cell-cycle phenotypes. MP and GO analysis of those upregulated phosphorylated proteins.

As a summary, the outcomes of the above analysis revealed that the strengthening of EPO receptor signaling and PluriNetWork by the administration of FG-4592, while the wakening of B cell proliferation and T cell proliferation pathways, for creating a better internal environment for hematopoietic cell implantation.

**Figure S5.** HIF-1α protein was stabilized *in vivo* upon co-administration of FG-4592 during the course of myeloablation, leading to an increase in survival rates of the myeloablated mice. **A.** HIF-1α expression in Lin⁺ and Lin⁻ cells following conditioning. Intracellular HIF-1α protein levels in the Lin^-^ or Lin^+^ cells were measured by flow cytometry, and a significant increase in expression of HIF-1α for those FG-4592-treated Lin^+^ cells was observed. The recipient mice were divided into two groups: one was treated with busulfan for 3 days, then with cyclophosphamide (BuCy) for another 2 days (Normal treatment group), while the other group was injected with FG-4592 intraperitoneally daily for 5 consecutive days during BuCy-based myeloablation. FG-4592 treatment group *vs*. normal treatment group, n=3, **P*<0.05. **B.** The survival curve of the mice after the normal treatment *vs.* the FG-4592 treatment. A much increase of the survival rate for those of FG-4592 treated mice was observed. The dosage of busulfan was shown in the figure 1A. Cyclophosphamide 200mg/kg, FG-4592 20mg/kg, n=10 for each group.

**Figure S6.** *In vitro* apoptosis of Lin⁻Sca-1⁺ and Lin⁻Sca-1⁻ cells following Bu/Cy ± FG-4592. (Detection of cell apoptosis after treating cells with demyelinating reagents in vitro for 24 hours). **A.** Apoptosis analysis of *Lin^-^Sca-1^+^* cells. **B.** Apoptosis analysis of Lin^-^Sca-1^-^ cells. **C.** Apoptosis analysis of Lin^+^ cells. Busulfan with FG-4592 vs. busulfan: **P*<0.01* or cyclophosphamide with FG-4592 vs. cyclophosphamide: ^##^*P<0.01*. n=3

**Figure S7.** Pathway map for apoptosis signaling of Normal treatment vs. WT

**Figure S8.** Pathway map for apoptosis signaling of FG-4592 treatment vs. WT

**Figure S9.** Pathway map for apoptosis signaling of FG-4592 treatment vs. normal treatment

**Figure S10.** FG-4592 promotes apoptosis-related signaling pathways during BuCy myeloablation: Inference and validation. **A.** Activation of MAPK and p53 apoptotic pathways. Two signaling pathways were inferred to be involved in FG-4592-mediated apoptosis promotion during myeloablation. The path included MAPK signaling and p53 signaling. **B.** Western blot validation in Lin⁻Sca-1⁺, Lin⁻Sca-1⁻, and BM-MNCs. Changes in signaling pathways during apoptosis in Lin^-^Sca-1^+^ hematopoietic cells, Lin^-^Sca-1^-^ non-hematopoietic cells, and total BM-MNCs were evidenced by Western blotting of phosphorated proteins.

**Figure S11.** The effect of HIF-PH inhibition or stabilization on the apoptosis of various types of BM-MNCs during *in vitro* myeloablation by Bu or Cy. **A.** The apoptosis ratio of the Lin^-^Sca-1^+^ cells. **B.** The apoptosis ratio of the Lin^-^Sca-1^-^ cells. ** vs. Bu+FG-4592，*P<0.01*; # vs. Bu+FG-4592，*P<0.05*；^##^ vs. Bu+FG-4592, *P<0.01*, n=3

Table S1. The outcomes of blood routine test on week 10 after low-dose BM-MNCs transplantation. The hematological parameters all returned to the normal levels (except for Plt levels which were lower than that of WT mice, but there was no difference in Plt levels between the two groups), indicating that FG-4592 treatment is effective, safe, and reliable.

| Group | RBC (10^12^/L) | HGB (g/L) | HCT (%) | WBC (10^9^/L) | PLT (10^9^/L) |
| --- | --- | --- | --- | --- | --- |
| WT | 10.2±1.0 | 158.8±14.3 | 52.4±3.7 | 11.2±0.8 | 1671.9±257.4 |
| Normal treatment | 9.8±1.5 | 157.7±14.9 | 51.6±4.8 | 12.1±1.9 | 1445.8±106.3* |
| FG-4592 treatment | 10.5±0.7 | 161.4±9.0 | 52.8±3.1 | 11.7±3.0 | 1416.3±218.7* |

Values represent mean ± SD; RBC: red blood cell; Hgb: hemoglobin; Hct: hematocrit; WBC: white blood cell; Plt: Platelet. Statistically significant differences were observed, Normal treated group (n=10) and FG-4592 treated group (n=10) compared
